# Effect of Dentin Conditioning with EDTA and Diode Lasers on Expression of Odontoblast-like Cell Markers of Dental Pulp Stem Cells

**DOI:** 10.3390/dj11090210

**Published:** 2023-09-04

**Authors:** Gabriela Martín, Valentín Preve, Kenneth Hargreaves, Anibal Diogenes, Carolina Inostroza, Nicole Saint-Jean, Claudia Brizuela

**Affiliations:** 1 Department of Endodontics, Universidad Católica de Córdoba, Córdoba 5000, Argentina; 2 Faculty of Dentistry, Universidad Nacional de Córdoba, Córdoba 5000, Argentina; 3 Private Practice, Montevideo 11000, Uruguay; 4 Department of Endodontics, University of Texas Health Science Center at San Antonio, San Antonio, TX 78229, USA; hargreaves@uthscsa.edu (K.H.); diogenes@uthscsa.edu (A.D.); 5 Dental School, Universidad de los Andes, Santiago de Chile 7550000, Chile; caroviriffo@gmail.com (C.I.); saintjean.nicole@gmail.com (N.S.-J.); cbrizuela@uandes.cl (C.B.)

**Keywords:** regenerative endodontics, stem cells, differentiation markers, diode laser, irrigation

## Abstract

Regenerative endodontic procedures rely on the delivery of mesenchymal stem cells into the root canal and on the effect of local growth factors from the dentin and blood clot. The aim of this study was to assess the effect of dentin conditioning with ethylenediamine tetraacetic acid (EDTA) and diode lasers with different wavelengths (808 nm and 980 nm) on the expression of odontoblast-like cell markers. Forty dentin cylinders were divided into four groups according to the irrigation protocol: EDTA, EDTA + 808 nm diode laser, EDTA + 980 nm diode laser, and phosphate-buffered saline as the control group. Dental pulp stem cells were seeded into the previously conditioned cylinders and incubated for 14 days. The quantitative real-time polymerase chain reaction was used to evaluate the expression of dentin sialophosphoprotein (DSPP), dentin morphoprotein-1 (DMP-1), and transforming growth factor-beta 1 (TGF-β1). Data analysis was performed using the Kruskal–Wallis test. The activation of EDTA with 980 nm and 808 nm diode lasers resulted in lower DSPP and DMP-1 expression than that for EDTA alone (*p* < 0.05 and *p* < 0.01, respectively). The expression of TGF was similar among all groups. The highest level of expression of odontoblast-like differentiation markers was observed with EDTA alone. However, the use of an 808 nm diode laser during EDTA irrigation reduced the expression of odontoblastic differentiation markers.

## 1. Introduction

A regenerative endodontic procedure (REP) is an alternative treatment for necrotic immature permanent teeth [1] and mature teeth with apical periodontitis [2]. This procedure is also known as the revascularization procedure because it suggests that the blood supply to the previously necrotic pulp space is required to achieve apical closure and increase the dentin wall thickness. Other authors prefer the term revitalization, since the new, vital tissue formed in the root canal, although not necessarily pulp tissue, comprises not only blood vessels but also connective tissue [3]. These procedures aim to replace damaged structures, including the dentin and the root structure, and rely on the delivery of mesenchymal stem cells from the periradicular area and on local morphogens found in the blood clot and radicular dentin [4]. 

Odontoblast-like cells may exhibit different cell phenotypes influenced by factors such as the source of progenitor cells and signaling events involved in their differentiation, which may depend on the availability of growth factors.

The dentin matrix has been recognized as an important reservoir of bioactive signaling molecules such as dentin matrix protein-1 (DMP-1), transforming growth factor-beta 1 (TGF-β1), vascular endothelial growth factor (VEGF), bone morphogenetic protein 2 (BMP-2), and fibroblast growth factor-2 (FGF2) [5]. These bioactive molecules induce cell growth and differentiation by binding to specific receptors.

Irrigation is crucial in REPs, playing a key role in root canal disinfection and dentin conditioning, which can have a significant impact on the ability of cells to attach to the radicular surface. High concentrations of sodium hypochlorite (NaOCl) reduce cell adherence to dentin [6], survival, and proliferation [7]; however, this effect is reversed with 17% ethylenediamine tetraacetic acid (EDTA) [8]. Dentin conditioning with a low concentration of NaOCl (1.5%) and EDTA (17%) might be favorable in REPs [9]. On the other hand, sodium thiosulfate is an irrigating solution that is employed to counteract the effect of NaOCl in root canal disinfection [10,11]. However, the indirect effect of a final rinse with 10% sodium thiosulfate on cellular activity, in root canals obturated temporarily with calcium hydroxide, calcium hypochlorite, and triple antibiotic pastes, has shown that the adverse effects caused by calcium hypochlorite paste on cell viability and mineralization activity cannot be neutralized with 10% sodium thiosulfate [12]. Additionally, various approaches suggest using sonic, ultrasonic, and laser conditioning as adjuncts in the chemical disinfection, to improve root canal irrigation. 

Since 1990, lasers have been used for dental applications with a focus on root canal disinfection in Endodontics. Alternative ways to activate irrigation involve high-power diode lasers and photoactivated disinfection, which have been proposed to enable access to areas that are not reachable with conventional techniques of root canal irrigation [13]. High-power diode lasers eliminate microorganisms by absorbing energy from the irrigating solution and generating heat. They have shown effectiveness in eradicating a wide range of microorganisms [14]. The near-infrared diode laser has demonstrated promising results due to its thin and flexible fiber, enabling access to root canals and enhancing dentin disinfection [15]. Many reports on high-power diode lasers concentrate on their antibacterial efficacy [16,17], smear layer removal from root canal walls [18], and the impact of lasers on the mechanical features of the dentin [19,20,21]. However, there is no evidence on the indirect effects of diode lasers on stem cells’ survival and differentiation gene markers. Thus, this study aimed to assess the effect of dentin conditioning with 808 nm and 980 nm diode lasers and EDTA on the expression of odontoblast-like cell markers.

## 2. Materials and Methods

### 2.1. Laser System

Two different gallium–aluminum–arsenide (GaAlAs) diode lasers were used: an 808 nm wavelength system equipped with a 7W power source (Medelux Medical Equipment Co., Ltd., Shanghai, China) and a 980 nm laser with 10W (Wuhan Gigaa Optronics Technology Co., Ltd., Wuhan, China). Both lasers were used with a 300 µm optical fiber to deliver the irradiation into the root canal during irrigation with EDTA solution.

### 2.2. Preparation of Dentin Cylinders

Forty-three molars diagnosed with normal pulp tissue were collected from adult donors of the Surgery Clinic at the Health Sciences Faculty of Universidad Catolica de Cordoba from Argentina; they were collected for reasons not related to the present study (Ethical Approval Code E20191015). The molars were promptly washed with sterile phosphate-buffered saline (1× PBS) and placed in a sterile PBS-filled tube for storage until root tip segments were obtained, following the methodology described in a previous study [9]. Subsequently, the samples were treated with iodine and 70% ethanol to minimize the risk of contamination and then washed five times with phosphate-buffered saline. The roots were sectioned into segment lengths of 5 mm. For instrumentation of the root canal, #130 LSX files (Sybron Endo, Orange, CA, USA) were used, and irrigation with sterile saline was performed. The dentin cylinders were sterilized using ethylene oxide gas and stored at room temperature. 

### 2.3. Isolation and DPSC Culture

The sample of dental pulp tissue was obtained from the extracted third molars from healthy donors at the Dental School, Universidad de los Andes, Chile. All donors accepted to participate in this study and signed an informed consent form. The protocol was evaluated and approved by the ethics committee of the University of the Andes, Santiago, Chile. Explants of the pulp were cultured on culture plates with complete media supplemented with alpha-MEM (Minimum Eagle Medium, Invitrogen), 10% fetal bovine serum (Fetal Bovine Serum, HyClone^®^), and 1% antibiotic (Penicillin–Streptomycin, PenStrep^®^, Invitrogen) at an incubator at 37 °C and 5% CO_2_. The medium was changed every 3 days until cellular migration outside of the explants was observed. The explants were removed, and the cells were cultured until a cellular confluence of 80% was achieved. At this point, the cells were removed from the plastic by treatment with 0.25% Trypsin/EDTA (GIBCO, Invitrogen), ending up in passage 0. The process was repeated until cells achieved passage 4, where functional characterization was performed. 

### 2.4. Immunophenotypical Profile by Flow Cytometry

For the immunophenotypic characterization, DPSCs at passage 4 were incubated with the following antibodies: CD105, CD90, CD73, CD34, CD45, CD19, and HLA-DR (BD, USA) for 20 min at 40 °C in a dark area. Then, they were washed with 4 mL of PBS 1× and centrifuged at 1800 rpm for 6 min, and the supernatant was removed. The data were collected using a FACS Canto II Flow cytometer (BD Biosciences, San Jose, CA, USA) and analyzed with FlowJo analysis software.

### 2.5. Differentiation Assays

The characterized passage 4 DPSCs with a confluence of 80% were cultured in 3 different culture plates in order to induce adipogenic, osteogenic, and chondrogenic differentiation (StemPro Differentiation Kits, Thermo Fisher Scientific TM). They were incubated at 37 °C and 5% CO_2_. DPSCs without induction media were cultured in parallel basal media. Images were captured with the phase contrast, inverted light microscope Olympus CKX41 (Tokyo, Japan).

#### 2.5.1. Osteogenic Differentiation

The dental pulp stem cells were cultured at a density of 35,000 cells/cm^2^ in 4-well plates. Upon reaching 100% confluence, 500 µL of a differentiation medium (StemPro^®^, Differentiation Kits, Thermo Fisher Scientific TM) was added. This medium included alpha-MEM, 10% FBS, 1% PenStrep^®^, 0.1 µM dexamethasone, 10 mM B-glycerolphosphate, 50 ug/mL ascorbate-2-phosphate. At 4 weeks, the differentiation was stopped, and the cells were stained with a 40 mM Von Kossa, 0.1 M NaH_2_PO_4_ solution, pH 4.3 (Sigma, St. Louis, MO, USA). The cells in plates were washed twice with PBS and fixed with 70% ethanol for 30 min at room temperature. The cells were then washed twice with PBS and stained with 40 mM Von Kossa for 10 min at room temperature. The cells were washed once again twice with 1× PBS and finally washed five times with bi-distilled water and visualized using a bright-field microscope (Olympus CKX41 Tokyo, Japan).

#### 2.5.2. Chondrogenic Differentiation

A cellular density of 30,000 dental pulp of stem cells in a volume of 10 µL as a microdrop was cultured on 4-well plates. The adherent cells were incubated with the differentiation kit medium (StemPro^®^ Differentiation Kits, Thermo Fisher Scientific TM), Alpha-MEM supplemented with 10% FBS, 1% PenStrep^®^, 0.1 µM dexamethasone, 5 µg/mL insulin, 10 ng/mL TGF-beta 1, and 50 µg/mL ascorbate-2-phosphate. After 2 weeks in culture, the cellular micro masses were verified, and the differentiation was stopped. The cells were stained with Safranin-O (Sigma, St. Louis, MO, USA), by preparing a 0.1% Safranin O solution. Finally, the cells were washed 5 times with 0.15 mL/cm^2^ absolute ethanol (100%) and 5 times with bi-distilled water and visualized using a bright-field microscope Olympus CKX41 (Tokyo, Japan).

#### 2.5.3. Adipogenic Differentiation

The dental pulp stem cells were cultured at a density of 25,000 cells/cm^2^ in 4-well plates. Upon reaching 100% confluence, 500 µL of a differentiation medium (StemPro^®^, Differentiation Kits, Thermo Fisher Scientific TM) was added. This medium comprised alpha-MEM culture medium supplemented with 10% FBS, 1% PenStrep^®^, 0.1 µM dexamethasone, 10 µg/mL insulin, 0.02 mg/mL indomethacin. At 4 weeks, the DPSCs were washed with 1× PBS and visualized using a bright-field microscope Olympus CKX41 (Tokyo, Japan).

### 2.6. Irrigation and Laser Application

The dentin cylinders were immersed in a sterile phosphate-buffered saline (PBS) solution for a minimum of 10 min to permit dentin rehydration. Subsequently, each cylinder was placed in a device and irrigated with an 18 G needle and 10 mL syringe.

According to the irrigating solution and laser application, the root segments were randomly assigned to four groups, each consisting of ten samples:

- 1: phosphate-buffered saline (PBS);
- 2: 17% EDTA;
- 3: 17% EDTA + 808 nm GaAlAs diode laser;
- 4: 17% EDTA + 980 nm GaAlAs diode laser.

Each sample was irrigated with 10 mL of 17% EDTA or PBS for 5 min. In both laser groups, the root canal was irrigated with 5 mL of 17% EDTA. Subsequently, the 808 nm or 980 nm GaAlAs laser diode system was applied, at 1.5 w (45 J/cm^2^), output continuous wave power settings during 6 s, 5 times in a continuous circular motion rotation movement with intervals of 30 s between irradiations. At the intervals, 1 mL of the irrigating solution was used before laser application to minimize the heating effect of the laser. Next, 5 mL of 17% EDTA was delivered into the root canal for 1 min. Finally, the residual irrigant was removed by irrigating all samples with a final flush of sterile saline (20 mL). 

The samples were dried with sterile paper towels before seeding the DPSCs in Matrigel BD (Bioscience, USA, cat. 354234) using the thick gel method as per the manufacturer’s instructions. The dentin cylinders were placed into 24-well plates. The root canals were then filled with 2 µL of PBS containing a total of 2.5 × 10^5^ DPSCs and 10 µL of Matrigel BD using a pipette. After the polymerization of the Matrigel BD for 30 min at 37 °C and 5% CO_2_, medium (described previously) was added to the samples, and they were incubated at 37 °C and 5% CO_2_ for 14 days.

### 2.7. Expression of Odontoblast-like Cell Markers 

The expression of transforming growth factor-beta 1 (TGF-β1), dentin morphoprotein-1 (DMP-1), and dentin sialophosphoprotein (DSPP) was evaluated using quantitative real-time reverse-transcription polymerase chain reaction (qRT-PCR). After culturing the DPSCs and performing the experiments, the culture medium from the dish was removed from the adherent cells. The cells were washed with PBS 1×, and once the cells were washed and prepared, RNAlater^®^ was added directly to the culture dish to cover the cells. Once the RNA was stabilized, the cells were stored at −80 °C, for long-term storage until the RNA extraction procedure. The RNA extraction process utilized the RNeasy Mini Kit (Qiagen, Valencia, CA, USA) to isolate the total RNA. Following that, any potential contamination from genomic DNA was eliminated via DNASE^®^ treatment utilizing the DNA-free kit (Ambion, Austin, TX, USA) in accordance with a standardized protocol. The total RNA samples were utilized as input in one-step, real-time polymerase chain reactions, employing the TaqMan One-Step RT-PCR Master Mix Kit (Life Technologies, Carlsbad, CA, USA) and the TaqMan gene expression assays for human DSPP (Hs00171962_m1), TGF-β1 (Hs00998133_m1), and DMP-1 (Hs01009391_g1) (Life Technologies).

### 2.8. Statistical Analysis

All experiments were performed in duplicate, and the results were expressed as the mean. The statistical analysis was conducted employing the Kruskal–Wallis test. A probability value of *p* < 0.05 (*) was considered statistically significant.

## 3. Results

### 3.1. Immunophenotypical Profile by Flow Cytometry 

**Figure 1:**
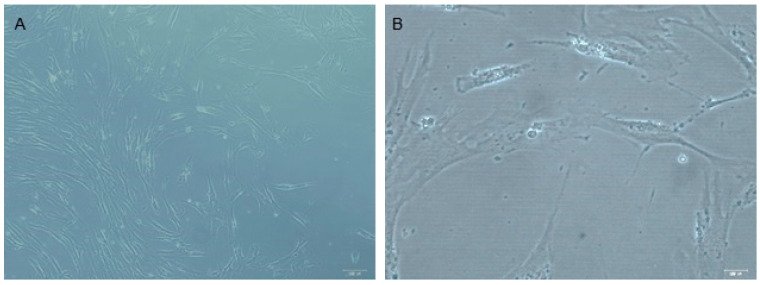
DPSCs in culture. (**A**) Microscopy photo at magnification 10× and (**B**) 40×. Bar = 100 Um. The DPSC characterization included morphology analysis in cell culture and monitoring of the fibroblastoid phenotype.

**Figure 2:**
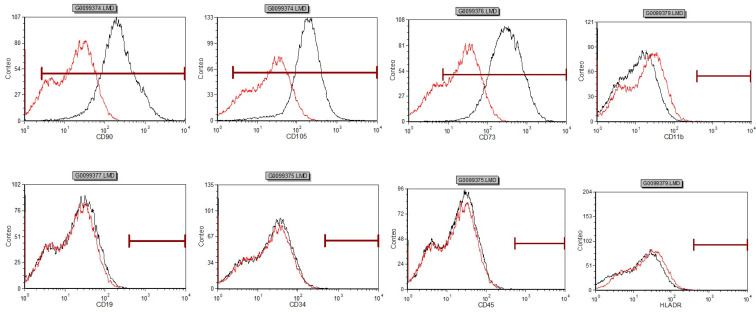
Stem cells from dental pulp coexpress CD90 (99.75%), CD105 (99.74%), CD73 (99.72%) in greater than 95% of the cells as detected by flow cytometry. Negative expression was observed for CD11b (0.01%), CD19 (0.01%), CD34 (0.21%), CD45 (0.01%), and (HLA-DR 0.88%).

The dental pulp stem cells displayed a fibroblast-like morphology in cell culture (Figure 1) and showed a high positive expression of the common MSC markers such as CD105 (99.74%), CD90 (99.75%), CD73 (99.72%). In addition, the cells had low expression of the negative control markers CD34 (0.21%), CD45 (0.01%), CD19 (0.01%), and HLA-DR (0.88%) (Figure 2). 

### 3.2. Differentiation Assays

**Figure 3:**
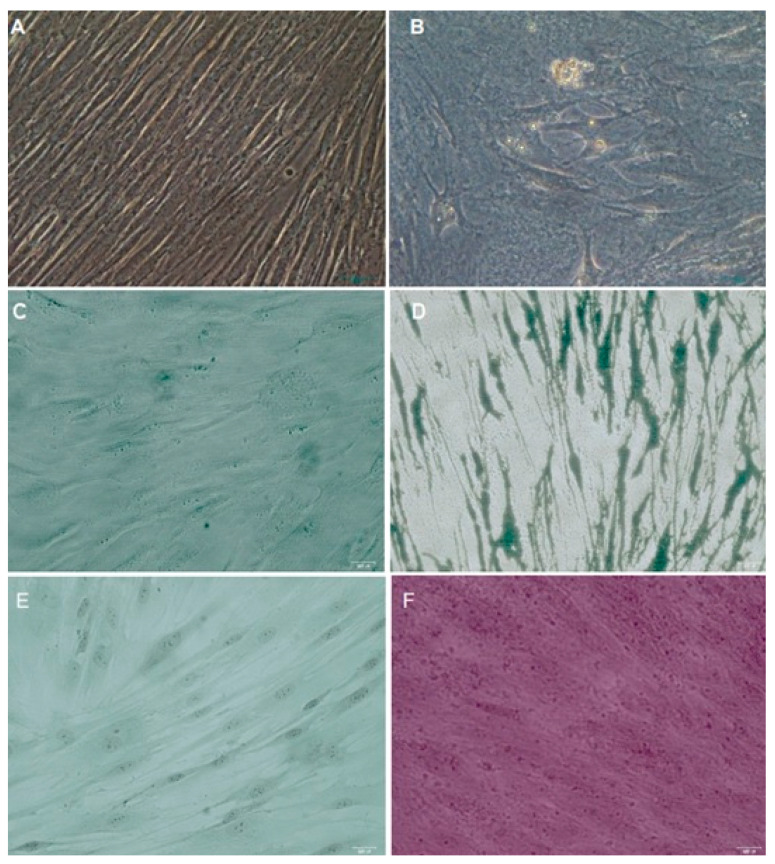
Differentiation potential showed that DPSCs form (**A**,**B**) adipocites, (**C**,**D**) osteoclasts, and (**E**,**F**) chondrogenic masses in culture conditions. Magnification 40×.

The dental pulp stem cells when cultured in a defined differentiation medium showed multipotency differentiation into osteogenic, chondrogenic, and adipogenic lineages in culture (Figure 3).

### 3.3. Expression of Odontoblast-like Cell Markers 

**Figure 4:**
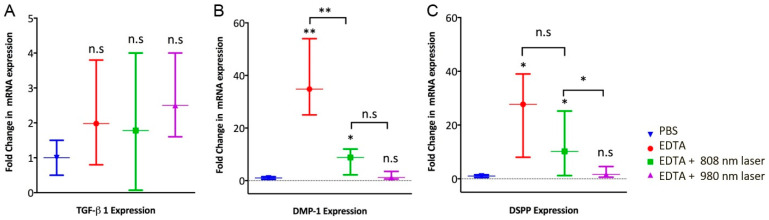
Expression of (**A**) transforming growth factor-beta 1 (TGF-β1), (**B**) dentin morphoprotein-1 (DMP-1), and (**C**) dentin sialophosphoprotein (DSPP) was determined by qRT-PCR. DPSCs (25,000 cells) were embedded in a Matrigel BD and spread into dentin cylinders previously irrigated with phosphate-buffered saline (PBS), 17% EDTA, and 17% EDTA activated with diode laser 808 nm and 980 nm, respectively. n.s not significant; * *p* < 0.05; and ** *p* < 0.01, as tested by Kruskal–Wallis test.

The control group treated with PBS revealed the lowest expression of all odontoblast-like cell markers. The group irrigated with EDTA promoted the highest level of DSPP and DMP1 expression but had no effect on the expression TGF-β1. The application of an 808 nm laser to 17% EDTA irrigation showed a lower expression of DSPP and DMP-1 comparable with that for the EDTA group (*p* < 0.01), but still higher than that for the 980 nm laser and the control group (*p*< 0.05). Dentin conditioning with a 980 nm laser resulted in the lowest level of DSPP and DMP-1 expression, among the experimental groups, with no statistically significant difference compared to the control group. The comparison between both laser groups indicated that employing the 808 nm laser resulted in a lesser impact on odontoblastic differentiation (DSPP, DMP-1) than that of the 980 nm laser group. Finally, the expression of TGF was similar among all groups (Figure 4). 

## 4. Discussion

Regenerative Endodontics is based on clinical and biological principles. Stem cells, growth factors, and scaffolds play an important role in inducing the generation of a new, vital tissue and a continuous root maturation [22]. Revascularization results are subject to numerous factors, including patient age, apex diameter, and root canal infection. Therefore, irrigation is crucial in REPs for the disinfection of the root canals [23], and EDTA has demonstrated the ability to enhance the viability and differentiation of stem cells [9]. Over the past few years, the use of laser-activated irrigating solutions has emerged as an alternative approach in endodontic therapy, with the potential to reduce bacteria and alter the root canal surface [18]. The findings of this study have notable clinical implications, indicating that dentin conditioning with EDTA alone leads to the highest expression of odontoblast-like cell markers. This observation suggests a potential enhancement in revascularization procedures. Nevertheless, caution is advised when utilizing 808 nm diode lasers in combination with EDTA, as it may lead to the reduced expression of odontoblastic differentiation markers.

In the realm of lasers, the choice among Gallium–Arsenide–Aluminum diode laser (GaAlAs), Neodymium: or Erbium:Yittrium–Aluminum–Garnet (Nd:YAG, Er:YAG), and Erbium–Chromium:Yittrium–Selenium–Gallium–Garnet lasers (Er,Cr:YSGG) often hinges on the specific requirements of dental applications. These lasers are commonly used for root canal irrigation, pulp tissue debridement, soft tissue incision and ablation, and subgingival soft tissue curettage. Furthermore, Er:YAG and Er,Cr:YSGG can be used for hard tissue conditioning and ablation. It is more readily absorbed by hydroxyapatite (found in teeth) than by water, making it more effective for dental procedures like cavity preparation and root canal treatments. For soft tissue procedures, it can be used effectively with proper settings to minimize thermal damage. The wavelength of the GaAlAs diode laser can vary from 650 to 810 to 980 nm. Hence, the current investigation aimed to assess whether the utilization of 808 nm and 980 nm GaAlAs diode lasers improved the effectiveness of EDTA in releasing bioactive molecules from the dentin matrix for odontoblastic differentiation. 

Different irrigation protocols have been published in numerous regenerative endodontic case reports with the aim of achieving maximal disinfection without taking into account their potential impact on stem cells. Maintaining a balance between disinfection and the establishment of an intracanal micro-environment is crucial for promoting the proliferation and differentiation of stem cells. Sodium hypochlorite is the most commonly used irrigating solution for chemical disinfection due to its bactericidal efficacy and tissue-dissolving ability. However, studies have shown that it can be harmful to stem cells due to its cytotoxic effects [24]. An in vitro investigation has indicated that the survival of Stem Cells from the Apical Papilla (SCAPs) was negatively affected by irrigation with 6% NaOCl, whereas 17% EDTA enhanced their survival rate [7]. The indirect effect of chemical solutions on the release of bioactive molecules, such as growth factors that induce cell differentiation, from the dentin has been recognized [25]. The use of high concentrations of NaOCl for dentin conditioning was found to have an adverse impact on the survival and differentiation of SCAPs, whereas 1.5% NaOCl followed by 17% EDTA improved both the survival and the differentiation of SCAPs. Irrigation with 17% EDTA in the final step reduced the adverse effect of NaOCl on the SCAPs [9]. It was demonstrated that high concentrations of NaOCl can cause the denaturation of growth factors derived from the dentin [26], whereas EDTA solubilizes these growth factors that are present in the dentin, leading to an increase in their bioavailability [27,28]. The present study showed that irrigation with 17% EDTA prior to seeding DPSCs into a standardized root canal space promoted a high level of DSPP and DMP-1 expression, agreeing with previous reports [8,9]. 

The dentin matrix is rich in bioactive molecules that possess potent cell signaling properties, including TGF-β1 and vascular endothelial growth factor (VEGF). The TGF-β family appears to comprise important molecules mediating the signaling of odontoblast differentiation [5]. Also, mesenchymal stem cells (MSCs) are known to produce TGF-β1 that may act through an autocrine and paracrine manner. In this study, the level of TGF-β1 expression within the cells was similar in the three experimental groups, not only when EDTA irrigation was performed but also when 808 nm and 980 nm diode lasers were applied with the EDTA solution. An in vitro study demonstrated that DPSCs, isolated and seeded onto the dentin surface treated with chemical photoactivation, differentiated into odontoblast-like cells, which extended a cytoplasmic process into the dentin tubules [29]. Similar findings showed that DPSCs seeded in a tooth slice model exhibited higher proliferation and greater expression of odontoblastic markers than those placed in a scaffold alone [30]. 

There are many lasers with different wavelengths whose mode of action is directly irradiating the tooth surface with light energy, causing a thermal reaction. Erbium lasers (Er: YAG) have been used in endodontic procedures for their antibacterial action but their emission wavelength is 2940 nm, resulting in an elevated temperature and it could be a disadvantage [31]. The photon-induced photoacoustic streaming (PIPS) is a low-level laser with a narrowed tip used to activate the root canal irrigation (20 mJ). PIPS generates a photoacoustic pressure wave in the irrigating solution, which eliminates the risk of thermal damage to the dentin structure [32]. The chemical changes in the root canal dentin depend on the type of laser wavelength and irrigating solution applied [19]. In the present study, the experimental groups were irrigated with 17% EDTA and the wavelength of low-level laser irradiation varied between 980 nm and 808 nm. The 980 nm wavelength laser showed a lower level of DSPP and DMP-1 expression than the 808 nm laser. Probably, this was due to the thermal heating of the environment that leads to dentin melting and the possible denaturation of the dentin matrix protein, including growth factors. The act of focusing laser pulses into a confined volume of fluid generates plasma, which leads to fast warming of the substance, causing an immediate expansion and the release of a shock wave. The application of laser to irrigating solutions causes absorption of the energy by the solution, creating bubbles in a liquid, which can implode and generate shock waves and acoustic cavitation on the surface [33]. In this study, when GaAlAs diode lasers were used, the potential heating and denaturation of growth factors or changes in the dentin surface may have contributed to the decrease in odontoblastic differentiation markers. This effect was more pronounced in the 980 nm laser group. 

The limitations of this study include a small sample size and limited marker evaluation, which may affect generalizability. The in vitro conditions may not fully represent the in vivo root canal environment. Future research should increase the sample size, evaluate more markers, and consider in vivo models.

Furthermore, future investigations should explore the optimal parameters for diode laser application and conduct in vivo studies to validate and extend these findings. Comprehensive evaluations of multiple cell markers and larger sample sizes will enhance understanding and applicability in regenerative endodontic protocols.

## 5. Conclusions

Incorporating 17% EDTA irrigation before seeding dental pulp stem cells into a standardized root canal space increased the expression of dentin sialophosphoprotein and dentin morphoprotein-1. However, when either 808 nm or 980 nm diode lasers were added to the 17% EDTA irrigation, the previously observed increase in dentin sialophosphoprotein and dentin morphoprotein-1 expression was reduced and eliminated, respectively. Interestingly, the expression of transforming growth factor-beta 1 remained consistent across all groups.

## Data Availability

Data is unavailable due to privacy.
